# Supplementary material for: Dynamics and spatial organization of Kv1.3 at the immunological synapse of human CD4+ T cells
Source: Biophys J. 2023 Aug 18;123(15):2271–81. doi: 10.1016/j.bpj.2023.08.011 (PMC11331042; doi:10.1016/j.bpj.2023.08.011)
Supplement: Document S1. Figures S1–S3 and Table S1 [file mmc1.pdf]

Biophysical Journal, Volume *123*

Supplemental information

Dynamics and spatial organization of Kv1.3 at the immunological synapse of human CD4+ T cells

Jesusa Capera, Ashwin Jainarayanan, María Navarro-Pérez, Salvatore Valvo, Philippos Demetriou, David Depoil, Irene Estadella, Audun Kvalvaag, James H. Felce, Antonio Felipe, and Michael L. Dustin

SUPPLEMENTAL FIGURES:

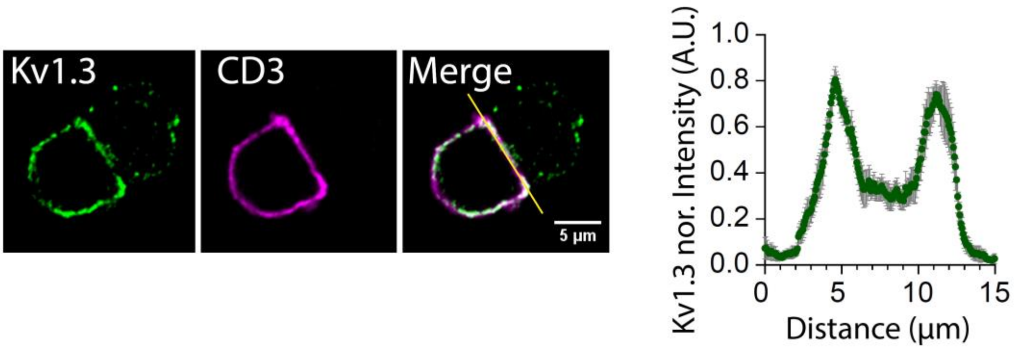

***Figure S1**. Kv1.3 accumulates as a distal ring in the case of T cell-APC conjugates*. Representative confocal image showing a conjugate between a human CD4+ T cell and a Raji B cell. Endogenous Kv1.3 and CD3 molecules were immunostained (green and magenta, respectively). A line (yellow in the merged image) was defined across the synaptic contact and Kv1.3 intensity. Plot shows the average Kv1.3 intensity across the line (n=10 cells).

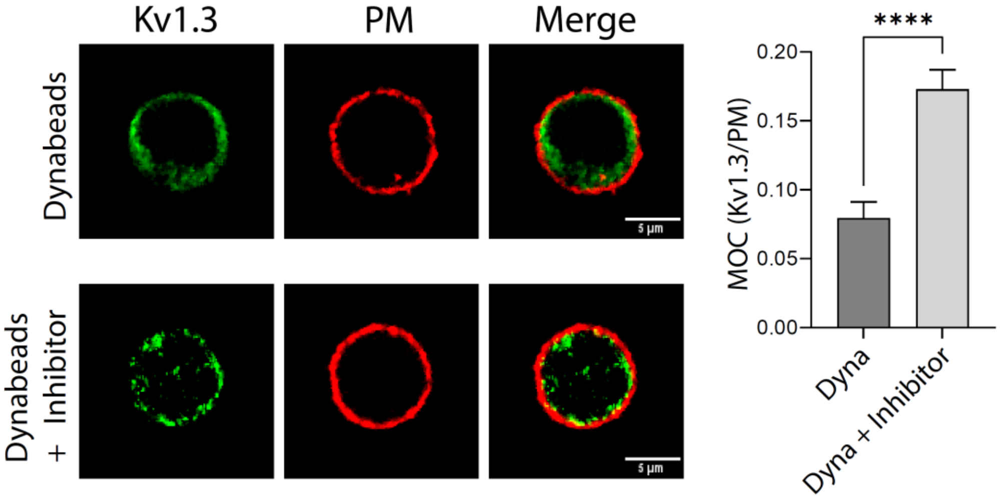

***Figure S2**. Kv1.3 internalization after activation*. Representative confocal images showing a a human CD4+ T cell after 15 min treatment with anti-CD3/CD28 Dynabeads (Gibco, 1:1 cell-to Dynabead ratio). Dynabead treatment was performed with or without 5 µg/ml of Chlorpromazine. Plot shows the Manders Coefficient between Kv1.3 and the plasma membrane marker. Data is the mean ± SE (n=30 cells, T-test).

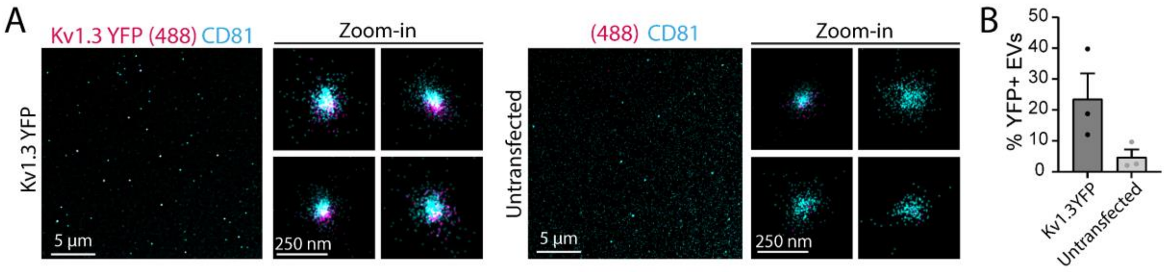

Figure S3. *Kv1.3 is released in extracellular vesicles (EVs) from activated CD4⁺ T cells.* **(A)** Representative dSTORM images showing EVs from activated CD4⁺ T cells transfected or not with Kv1.3-YFP and immunostained for CD81 (EV marker). Zoom in images show representative examples of individual EVs. **(B)** Quantification of the percentage of YFP positive EVs. EVs were segmented using the CD81 signal and YFP mean intensity was quantified for each EV. The mean YFP intensity for the untransfected sample was used to threshold for positive YFP particles. Data is the mean ± SE (n=3).

	Raw Intensity		Log10 transformed Intersity	
	Resting EVs	Activated EVs	Resting EVs	Activated EVs
KCNA1	31121	34121	4.493053544	4.533021751
KCNA2	21531	32193	4.333064201	4.50776145
KCNA3	1053100	827300000	6.022469613	8.917663024
KCNA4	21320	21231	4.3287872	4.32697045
KCNA5	17411	19821	4.240823716	4.297125562
KCNB1	12311	11311	4.090293331	4.053501002
KCNC1	14130	11130	4.150142162	4.046495164
KCND1	11643	13243	4.066064897	4.121986379
KCNS1	11212	14402	4.049683089	4.158422807
KCNS2	21113	22311	4.324549948	4.348519036
KCNAB2	13920000	246370000	7.143639235	8.391587824
KCNN4	1630600	19324000	6.212347438	7.286097029
TCRA	1	98024000	0	7.991332421
TCRB	1	30443000	0	7.483487448
CD3D	1805200000	3768200000	9.256525325	9.576133945
CD3E	4244000000	1.0516E+10	9.627775375	10.02185058
CD3G	1185700000	2535600000	9.07397482	9.404080743
CD4	1920700000	5881600000	9.283459536	9.769495485
CD40	43670000	1	7.640183192	0
CD40LG;TNFSF5	104820000	1283200000	8.020444155	9.108294351
CD81	3174400000	7498300000	9.50166165	9.874962812
TSG101	79443000	138660000	7.900055636	8.141951196

Table 1. Intensities (raw and log10 transformed values) of the molecules present in 1 ml of (10e8 EV/ml) of purified EVs from resting and activated CD4+ T cells.
