# Supplementary material for: Dynamics and spatial organization of Kv1.3 at the immunological synapse of human CD4+ T cells
Source: Biophys J. 2023 Aug 18;123(15):2271–81. doi: 10.1016/j.bpj.2023.08.011 (PMC11331042; doi:10.1016/j.bpj.2023.08.011)
Supplement: Document S2. Article plus supporting material [file mmc6.pdf]

Biophysical Journal
Article

Biophysical Society

Dynamics and spatial organization of Kv1.3 at the immunological synapse of human CD4+ T cells

Jesusa Capera,[1,2] Ashwin Jainarayanan,[1] María Navarro-Pérez,[2] Salvatore Valvo,[1] Philippos Demetriou,[1,3] David Depoil,[1] Irene Estadella,[2] Audun Kvalvaag,[1,4] James H. Felce,[1] Antonio Felipe,[2,*] and Michael L. Dustin[1,*]
[1]The Kennedy Institute of Rheumatology, Nuffield Department of Orthopaedics Rheumatology & Musculoskeletal Sciences, University of Oxford, Oxford, United Kingdom; [2]Molecular Physiology Laboratory, Departament de Bioquímica I Biomedicina Molecular, Institut de Biomedicina (IBUB), Universitat de Barcelona, Barcelona, Spain; [3]The Center for the Study of Haematological and Other Malignancies, Nicosia, Cyprus; and [4]Department of Molecular Cell Biology, Institute for Cancer Research, Oslo University Hospital, Oslo, Norway

ABSTRACT Formation of the immunological synapse (IS) is a key event during initiation of an adaptive immune response to a specific antigen. During this process, a T cell and an antigen presenting cell form a stable contact that allows the T cell to integrate both internal and external stimuli in order to decide whether to activate. The threshold for T cell activation depends on the strength and frequency of the calcium (Ca^{2+}) signaling induced by antigen recognition, and it must be tightly regulated to avoid undesired harm to healthy cells. Potassium (K^+) channels are recruited to the IS to maintain the negative membrane potential required to sustain Ca^{2+} entry. However, the precise localization of K^+ channels within the IS remains unknown. Here, we visualized the dynamic subsynaptic distribution of Kv1.3, the main voltage-gated potassium channel in human T cells. Upon T cell receptor engagement, Kv1.3 polarized toward the synaptic cleft and diffused throughout the F-actin rich distal compartment of the synaptic interface—an effect enhanced by CD2-CD58 corolla formation. As the synapse matured, Kv1.3 clusters were internalized at the center of the IS and released in extracellular vesicles. We propose a model in which specific distribution of Kv1.3 within the synapse indirectly regulates the channel function and that this process is limited through Kv1.3 internalization and release in extracellular vesicles.

SIGNIFICANCE During T cell activation through an immunological synapse (IS), potassium efflux sustains calcium influx, which is an essential secondary messenger. Our work describes the temporal and spatial organization of Kv1.3, the main voltage-gated potassium channel at the IS of $CD4^+$ T cells. Kv1.3 forms a distal ring at the IS and accumulates in the CD2-CD58 corolla, which is an emerging key regulator of T cell activation and sustains T cell receptor-dependent calcium fluxes. Over time, Kv1.3 action is spatially terminated by its recruitment to the center of the IS, where a dual mechanism takes place to limit Kv1.3 surface abundance: internalization and release in extracellular vesicles.

INTRODUCTION

T cell receptor (TCR) engagement by antigen presentation results in a stable contact between the T cell and the antigen presenting cell (APC) or target cell known as the immunological synapse (IS). Several types of receptors and effectors are dynamically organized at the synapse interface to integrate chemical, mechanical, and electrical signals that, if appropriate, will lead to T cell activation. The IS is compartmentalized into concentric supramolecular activation clusters (SMACs): the central SMAC (cSMAC), where the TCR and co-receptors accumulate after interaction with peptide-major histocompatibility complexes (pMHCs), the peripheral SMAC (pSMAC), which forms an adhesion ring enriched with LFA1 -ICAM1 interactions, and the distal SMAC (dSMAC), where TCR-pMHC microclusters originate and CD2-CD58 interactions accumulate in a corolla of close contacts domains (1,2).

Formation of TCR-pMHC microclusters triggers activation of phospholipase Cϒ (PLCϒ). Consequent increase of IP3 causes the release of Ca^{2+} from the endoplasmic reticulum (ER) lumen through the IP3R Ca^{2+} channel. This generates a small and local increase in cytosolic Ca^{2+}, which is not enough to activate gene expression. However, depletion of Ca^{2+} in the ER lumen induces activation of calcium release activated channels (CRACs), which will mediate a massive Ca^{2+} influx from the extracellular space. At this moment, the cytosolic Ca^{2+} concentration will reach the micromolar range and, if sustained long enough, lead to T cell activation (3,4).

Submitted May 12, 2023, and accepted for publication August 14, 2023.
*Correspondence: afelipe@ub.edu or michael.dustin@kennedy.ox.ac.uk
Editor: Diane Lidke.
https://doi.org/10.1016/j.bpj.2023.08.011
© 2023 Biophysical Society.
This is an open access article under the CC BY license (http://creativecommons.org/licenses/by/4.0/).

K^+ efflux is required for sustained Ca^{2+} influx, and K^+ channels are thus essential regulators of Ca^{2+} signaling. Two main K^+ channels are expressed on T cells, the Ca^{2+}-activated K^+ channel KCa3.1 and the voltage-gated K^+ channel Kv1.3. The membrane potential (ΔVm) for a resting T cell is approximately −50 mV, and Kv1.3 is an outward delayed-rectifier channel activated by membrane depolarization with half-activation at −30 mV. Therefore, these two channels open either when cytosolic Ca^{2+} concentration is increased (KCa3.1) or when the ΔVm is depolarized (Kv1.3). Thus, the negative membrane potential is maintained and Ca^{2+} influx sustained thanks to the activity of these two channels at the IS (5,6). Kv1.3 and KCa3.1 cooperate to mediate the immune response, and their relative abundance and localization must be precisely regulated during all stages of the T cell lifetime (7-9). Not surprisingly, dysregulation of the expression of these channels leads to severe immune defects. Kv1.3 is upregulated by autoreactive effector memory T cells, thus making this channel a promising therapeutic target against autoimmune disorders (10-12). For instance, Kv1.3 is highly expressed by autoreactive T cell clones in patients with multiple sclerosis or type 1 diabetes (13). It is also highly expressed in other autoimmune diseases, like systemic lupus erythematosus infiltrates (14), psoriatic skin lesions (15), and synovial infiltrates of patients with rheumatoid arthritis (16). Pharmacological inhibition of Kv1.3 ameliorates the prognosis in animal models of these diseases, and clinical trials show promising results in humans (14,15). Interestingly, the compartmentalization of Kv1.3 channels at the IS is altered in some autoimmune processes (17).

Accumulation of Kv1.3 at the IS takes place during the first minutes of the contact (17,18) and mainly occurs by early lateral movement of resident plasma membrane channels (7). In addition, mechanisms controlling the forward trafficking of the channel can also alter its recruitment to the IS. For example, PSD-95 interacts with Kv1.3 and favors its polarization toward the IS (18), whereas the negative regulatory subunit KCNE4 interacts with Kv1.3 and retains the channel in the ER, impairing the channel targeting to the IS and therefore causing a reduction in T cell proliferation and IL-2 production (19).

However, no information about the spatial organization and dynamic regulation of Kv1.3 within the SMAC compartments is available. In this work, we visualized and quantitatively analyzed the distribution of synaptic Kv1.3 with high spatial and temporal resolution.

MATERIALS AND METHODS

Isolation of primary human $CD4^+$ T cells and generation of effector $CD4^+$ T cells

Primary human $CD4^+$ T cells were isolated from anonymized leukopoiesis products provided by UK National Health Service Blood and Transplant, using the negative selection kits RosetteSep Human $CD4^+$ T Cell Enrichment Cocktail (STEMCELL Technologies). Human T cells were cultured at 37°C, 5% CO_2, in RPMI 1640 medium (Life Technologies) supplemented with 10% FCS, 4 mM L-glutamine, 1% penicillin-streptomycin (Gibco), 1% Non-Essential Amino Acids Solution (Thermo Fisher Scientific), and 10 mM HEPES (Life Technologies). T cell effector cells were generated by stimulation with anti-human CD3/CD28 Dynabeads (Gibco) for 3 days in the presence of 50 U/mL of IL-2. Dynabeads were then removed by magnetic separation, and cells were kept with supplemented RPMI containing 50 U/mL of IL-2 until day 7, at a density of 1–2 x 10^6 cells/mL.

Expression plasmids and mRNA electroporation

T.C. Holmes (University of California, Irvine, CA) provided the rat Kv1.3 in the pRcCMV construct, which was subcloned into pEYFP-C1 (Clontech). For mRNA electroporation, Kv1.3YFP was subcloned from pEYFP-C1 plasmid into pcDNA3 with a T7 promoter. Constructs were verified by sequencing. mRNA samples were prepared from linearized pcDNA3 plasmids. In vitro RNA transcription and in vitro poly-adenylation were performed using the mMESSAGE mMACHINE T7 ULTRA Transcription kit (Thermo Fisher Scientific), following manufacturer's instructions. For electroporation, cells were washed three times in Opti-MEM media (Life Technologies) and resuspended at 2–2.5 x 10^6 cells per 100 μL and mixed with 5 μg of the mRNA stock. Samples were then transferred to a Gene Pulser/Micropulser electroporation cuvette, 0.2 cm gap (Bio-Rad) and pulsed at 300 V for 2 ms in an ECM 830 Square Wave Electroporation System (BTX). Cells were immediately transferred to supplemented RPMI and cultured at 37°C, 5% CO_2 before usage after 18–24 h.

Preparation of supported-lipid bilayer and immunocytochemistry

Supported-lipid bilayers (SLBs) were prepared as previously described (20). Briefly, glass coverslips (Nexterion) were plasma cleaned and mounted onto six-channel chambers (Ibidi). Small unilamellar liposomes were prepared using 1,2-dioleoyl-sn-glycero-3-phosphocholine (Avanti Polar Lipids) supplemented with 12.5% 1,2-dioleoyl-sn-glycero-3-[(N-(5-amino-1-carboxypentyl) iminodiacetic acid) succinyl]-Ni (Avanti Polar Lipids). Channels in Ibidi chamber were covered with liposome mixture, blocked, and washed. SLBs were then incubated with the indicated mix of His-tagged proteins to achieve the desired density of molecules on the SLB: UCHT1 (30 molecules/μm^2), ICAM1 (200 molecules/μm^2), CD58 (200 molecules/μm^2), and CD80 (200 molecules/μm^2). Proteins were produced in house as complete extracellular domains with a C-terminal 12-His tag (ICAM-1, CD80, and CD58) or 14-His tag (UCHT1 Fab) expressed by Expi293F cells and purified by Ni2+ affinity chromatography and gel filtration. When indicated, ICAM-1 was labeled with Alexa Fluor 405 NHS ester, and the other proteins were labeled using Alexa Fluor dye-maleimide. For live imaging, cells were exposed on the SLB at 37°C and immediately imaged. For fixed cells, cells were exposed to the bilayers at 37°C for the indicated amount of time and fixed with 4% PFA and washed. For immunocytochemistry, cells were then permeabilized, blocked, and incubated with the primary antibody of interest (anti-clathrin) for 1 h. Next, samples were washed and incubated with the secondary antibody conjugated with AlexaFluor 647.

TIRF imaging, Airyscan confocal microscopy, dSTORM, and image analysis

Total internal reflection fluorescence (TIRF) imaging was performed on an Olympus IX83 inverted microscope with a TIRF module. The instrument was equipped with an Olympus UApON 150x TIRF N.A 1.45 objective,

405-, 488-, 568-, and 640-nm laser lines and Photomertrics Evolve delta EMCCD camera. For live imaging experiments, SLBs were transferred to a preheated incubator on top of the TIRF microscope and cells were added in the well. Airyscan imaging was performed using a Zeiss 980 LSM using a 63x oil 1.40 NA objective. Images were visualized using ZenBlue Software or Fiji (ImageJ). Image analysis was performed using Fiji (ImageJ). Pearson correlation coefficients were calculated using the JACoP plugin (21). Radial averages were generated as previously described (22). Single-particle tracking (SPT) analysis was performed using the TrackMate plugin v7.6.1 (23) on videos captured at 0.3 s/frame for 1 min. Briefly, particles were detected with an estimated diameter of 0.3 μm. Frame-to-frame particle linking was performed using a Linear Assignment Problem tracker, with a maximum linking distance of 0.3 μm, a gap-closing maximum distance of 0.4 μm, and a gap-closing maximum frame gap of one frame. Trajectory types were classified using the TraJClassifier plugin (24). For Ca^{2+} imaging, cells were incubated with Fura-2 (Life Technologies) for 20 min, washed, and exposed to the SLB for immediate imaging with the Olympus TIRF microscope in the widefield mode at 37°C. The emission spectra of Fura-2 dye was recorded when excited at 340 and 380 nm at 5-s intervals for 20 min on a Hamamatsu ORCA flash 2.8 version C11440-10C. Analysis of Ca^{2+} fluxes was performed as follows: cells were tracked using the TrackMate plugin in Fiji. Then, data were formatted for further analysis with MATLAB to identify and align the Ca^{2+} profiles on the maximum peak found within the first frames of a Ca^{2+} change response. Finally, individual calcium profiles were averaged for plotting. For dSTORM imaging, samples were mounted with a reducing buffer system and 10,000 images captured on a Nanoimager (Oxford Nanoimaging) with 100x oil-objective lens and analyzed with Nanoimager Software v1.4.8740 (Oxford Nanoimaging).

Isolation and purification of extracellular vesicles

Cells were incubated in serum-free medium for 48 h followed by collection of medium. To purify the extracellular vesicles (EVs) from T cell culture media, a series of procedures were performed. Initially, the medium underwent two rounds of centrifugation at 300 x *g* for 5 min to separate the supernatant, which effectively eliminated cells and cell debris. After this, a centrifugation step at 4000 x *g* for 10 min was carried out to isolate a purified supernatant, eliminating larger vesicles, including certain apoptotic bodies. Further, the purified supernatant was concentrated using Amicon centrifugal filter units with a molecular weight cutoff of 100 kDa, resulting in a volume reduction from 15 mL to 2 mL. The concentrated solution was then loaded onto a Sepharose 4 Fast Flow resin column (10 mm × 300 mm; GE Healthcare, Little Chalfont, UK), which was connected to the ÄKTA pure system (GE Healthcare). Using PBS as the eluent, the column was eluted at a flow rate of 0.5 mL/min while recording a chromatogram by measuring absorbance at 280 nm. Fractions of 0.5 mL were collected, and those containing SELC EVs (specific EVs of interest) were combined by pooling them together. To further concentrate the pooled fractions, Amicon centrifugal filter units with a molecular weight cutoff of 10 kDa were used, resulting in a final volume of 2 mL.

Proteomic analysis of isolated extracellular vesicles

The EV samples were reduced with 5 μL of 10 mM tris(2-carboxyethyl) phosphine and alkylated with 50 mM of iodoacetamide for 30 min each, then acidified with 12% phosphoric acid 10:1 vol:vol, and then transferred to S-trap columns. Then, samples were precipitated using 1:8 vol:vol dilution of each sample in 90% methanol in 100 mM triethylammonium bicarbonate and digested with trypsin (Promega, #V1115) overnight at 37°C. The samples were then run on an LC-MS system composed of an Evosep One and Bruker timsTOF Pro. For proteomic analyses, the raw files were searched against 201,801,131 entries in the Uniprot Homo sapiens database using MaxQuant version 1.6.10.43. The intensities were baselined using NTA concentration, and average-based normalization was carried out to obtain the enriched set of proteins in each EV sample.

Statistical analysis

All statistical tests were performed using GraphPad Prism 9 software. Student's *t*-test, one-way ANOVA, and Tukey's post hoc tests were used for statistical analysis, and $p < 0.05$ was considered statistically significant. All data are representative from at least three independent human blood donors.

RESULTS

Kv1.3 polarizes to the IS of human $CD4^+$ T cells

Kv1.3 is recruited to the IS (7,25). However, details on the subsynaptic arrangement of the channel within the SMAC compartments are missing, leading to an incomplete understanding of the channel function and regulation during early T cell activation. Here, we used an SLB system to mimic the surface of an APC. The advantage of this system is that it allows full control of the T cell activation and an optically ideal view of the entire process. Effector human $CD4^+$ T cells were electroporated with in-frame *KCNA3*-YFP encoding mRNA and exposed to the SLBs containing anti-CD3, ICAM1, CD80, and CD58 molecules (Fig. 1 *A*). Unless otherwise specified, all experiments in this work were performed using this SLB composition. After 15 min of SLB exposure, cells were fixed and imaged by Airyscan microscopy. Kv1.3 was detected mainly at the surface of the T cell as indicated by equatorial views (Fig. 1 *B*), with a clear polarization toward the contact with the SLB (Fig. 1 *B–D*; Video S1). However, the distribution of the channel was not homogeneous across the synaptic interface. Instead, Kv1.3 was concentrated in the dSMAC and the cSMAC (with anti-CD3). Central accumulation of the channel was also observed in intracellular compartments adjacent to the cSMAC (Fig. 1 *C* and *D*).

Kv1.3 organizes as a dynamic outer ring at the mature IS

To further characterize the distribution of Kv1.3 within the different SMAC compartments, we imaged the T cells activated on SLBs using TIRF microscopy. Cells were fixed and imaged after 15 min of SLB exposure. We visualized anti-CD3, ICAM1, and close contact areas defined by interference reflection microscopy (IRM) to identify the cSMAC, pSMAC, and dSMAC, respectively (Fig. 2 *A* and *B*). Synaptic Kv1.3 primarily localized to the dSMAC and partially overlapped with the cSMAC (Fig. 2 *B*). Next, we analyzed the dynamic behavior of synaptic Kv1.3 using live TIRF imaging. Mature synapses (SMAC compartments fully formed) were imaged in time-lapses at 0.3 s/frame for 1 min. Because Kv1.3 was highly dynamic at the synaptic

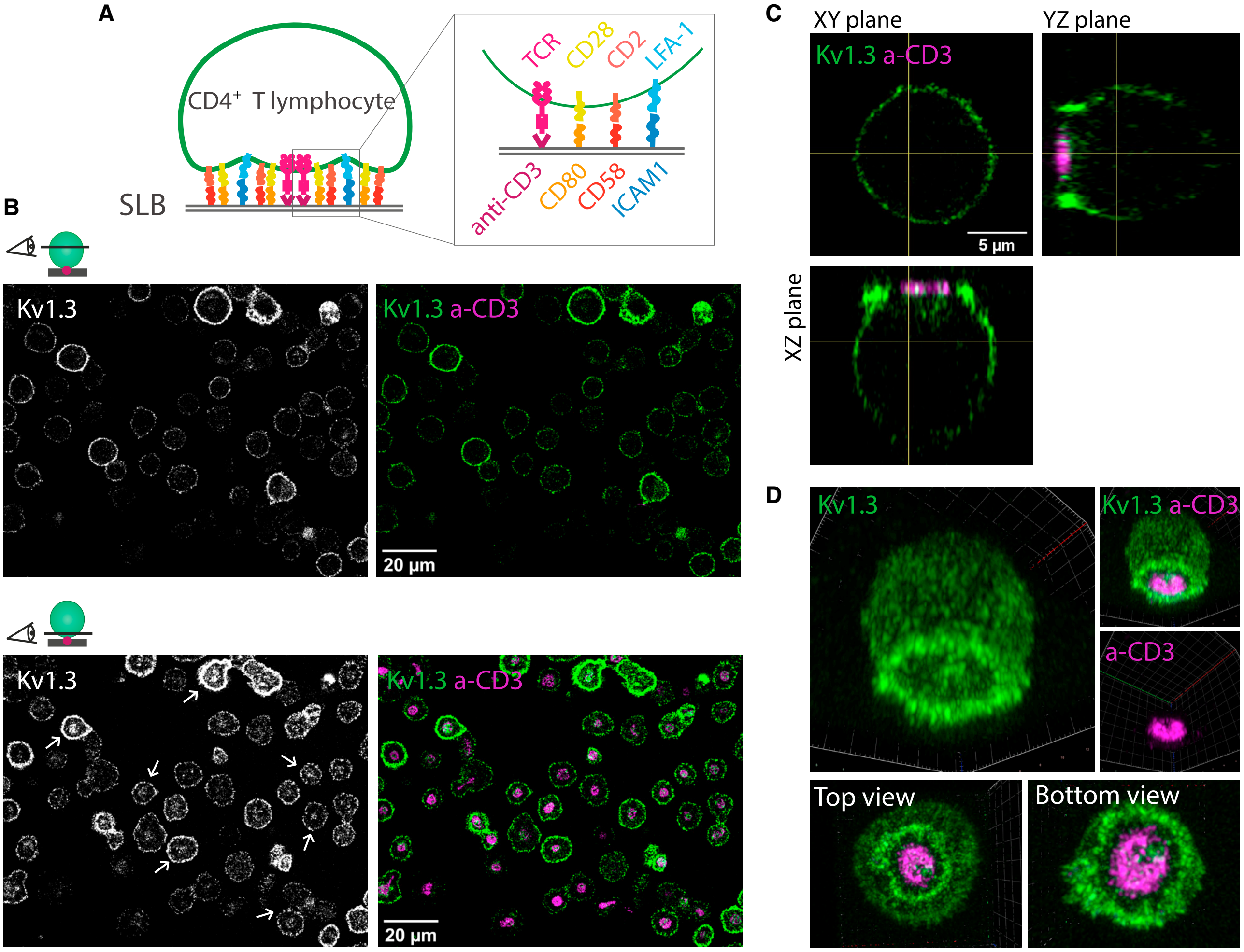

FIGURE 1 Kv1.3 tri-dimensional architecture at the immunological synapse. (*A*) Schematic representation of the supported-lipid bilayer (SLB) system used. Human CD4+ T cells expressing Kv1.3 YFP were exposed to SLBs containing anti-CD3 (UCHT1), CD80, CD58, and ICAM1. (*B*) Representative frames from a 3D Airyscan stack of human CD4+ T cells forming synapses with the SLB and fixed after 15 min of SLB exposure. a-CD3 proteins (*magenta*) were used to define the bilayer plane. Top images show a central frame in the 3D stack (approx. 4.5 μm from the bilayer). Bottom images show the bilayer plane. Arrows show the accumulation of Kv1.3 at the bilayer proximity. (*C*) Orthogonal views of a single cell forming a synapse with the SLB. Note the accumulation of Kv1.3 (*green*) at the SLB contact and the intracellular accumulation of Kv1.3 at the proximity of the cSMAC (a-CD3, *magenta*). (*D*) 3D view of the cell in (*C*) using maximal intensity projection. To see this figure in color, go online.

interface, we merged the frames into 2D time projections to obtain a representative snapshot of the temporal Kv1.3 distribution (Fig. 2 *C*; Video S2). We then analyzed the projections by radial averaging, as previously described (22), which confirmed that Kv1.3 mainly accumulated in the dSMAC (Fig. 2 *D*). We further confirmed the accumulation of Kv1.3 as a distal ring in T cell-APC conjugates (Fig. S1). Formation of the distal Kv1.3 ring was synapse dependent because it was only observed when the bilayers contained anti-CD3. Instead, when the bilayer contained only ICAM1, Kv1.3 distributed homogeneously with no central exclusion in the SLB contact over time (Fig. 3 *A*). Addition of CD80 alone or CD80 and CD58 to the SLB with ICAM1 and anti-CD3 did not change the gross localization of Kv1.3 (Fig. 3 *A*), but central exclusion of Kv1.3 was greatest with bilayers containing the full complement of costimulatory ligands (Fig. 3 *B*). This was also the condition that gave rise to the most sustained Ca^{2+} flux (Fig. 3 *C*).

Kv1.3 dynamics is dependent on its subsynaptic localization

Kv1.3 is naturally expressed on activated T cells at less than 1000 tetrameric channels per activated T cell (26,27). The mRNA electroporation approach allowed us to introduce fluorescent labels into the Kv1.3 tetramers while preserving the physiological density of <1 molecule/μm^2, which allows application of SPT with TIRF illumination and an EM-CCD detector. Synaptic Kv1.3 were highly dynamic. However, their kinetic behavior differed according to which SMAC compartment they resided in. Rapidly moving Kv1.3

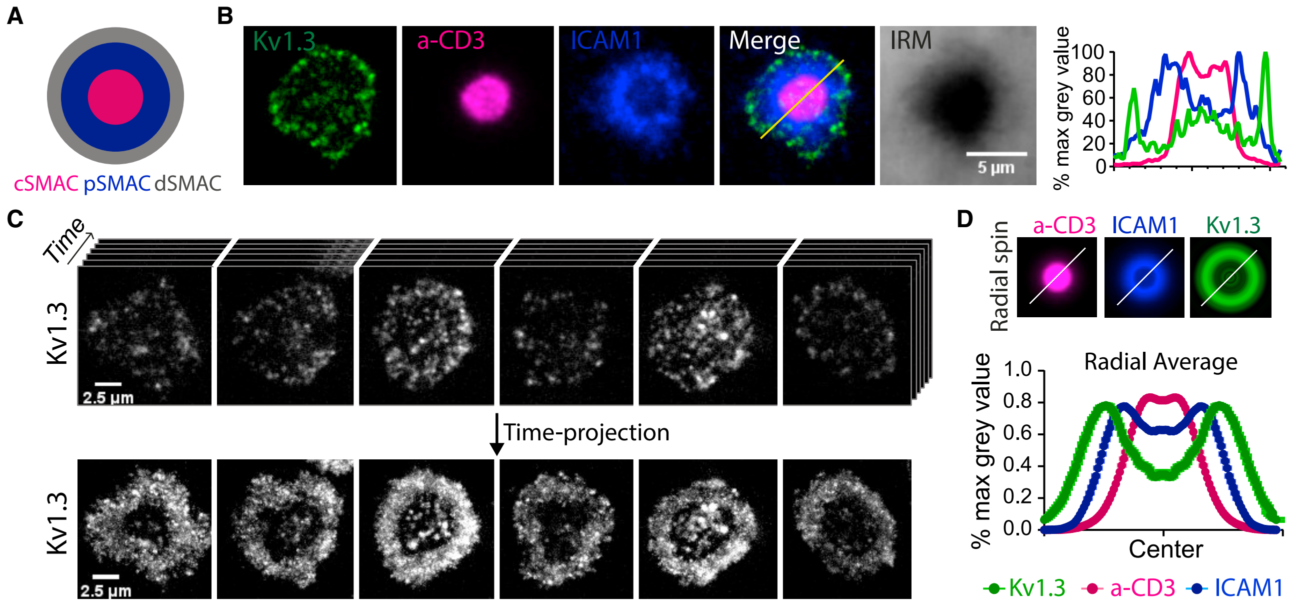

FIGURE 2 Kv1.3 organizes as a dynamic ring at the mature synapse platform. (*A*) Cartoon representing the bulls-eye view of a synapse organized in SMAC compartments (c, central; p, peripheral; d, distal). (*B*) Representative TIRF image of a CD4+ T cell forming a synapse with an SLB. a-CD3 (*magenta*) accumulates at the cSMAC, and ICAM1 (*blue*) forms a ring at the pSMAC. Contrast (IRM) image shows the surface of the cell-SLB contact. The cell expressed Kv1.3-YFP (*green*). The SLB also contained unlabeled CD80 and CD58. The histogram on the right shows the line intensity profile. (*C*) Top: representative TIRF images from videos of live CD4+ T cells expressing Kv1.3 YFP (*gray*) and forming synapses with an SLB. Videos were recorded after less than 15 min of bilayer exposure, when the cells were forming mature synapses (as identified by the presence of ICAM1 rings). Videos were recorded at 0.3 s/frame for 1 min. Bottom: maximal intensity projection over time (time projection) from the videos, to summarize the distribution of Kv1.3 over time. Note that Kv1.3 mostly accumulates in a peripheral ring with some presence at the center of the synapse. (*D*) Top: representative radial spin images used to quantify the radial average of the intensity for each protein. Bottom: histogram showing the average radial distribution of a-CD3, ICAM1, and Kv1.3. Data are the mean ± SE. Single frame images were used for a-CD3 and ICAM1 ($n > 200$ cells), and single-frame time projections were used for Kv1.3 for a more representative distribution of the channel at the synapse ($n > 60$ cells). To see this figure in color, go online.

were located in the dSMAC (Fig. 4 *A*). Whereas, slower moving Kv1.3 clusters were mostly located in the cSMAC (Fig. 4 *A*). To analyze this behavior, we performed SPT analysis of synaptic Kv1.3. We used time projections of the image sequences to define the Kv1.3 territories in the dSMAC and the cSMAC. Kv1.3 distal tracks versus central tracks were compared. The analysis confirmed that central tracks were more static, showing a significantly reduced speed, displacement, and confinement ratio compared with the distal Kv1.3 tracks (Fig. 4 *B*). In addition, Kv1.3 tracks were classified based on their type of trajectory (Fig. 4 *C*). The data confirmed that central Kv1.3 tracks were more likely to be confined or subdiffusing than the distal Kv1.3 tracks, which tended to display a more active/directed motion (clusters) or normal diffusion behavior.

Kv1.3 preferentially accumulates at the CD2/CD58 corolla

Next, we interrogated the functional correlates of the central and distal Kv1.3. The CD2-CD58 complex is known to form a corolla within the dSMAC. This structure boosts TCR-proximal signaling (2). As shown above, the presence of CD80 and CD58 on the SLB simultaneously enhanced formation of the Kv1.3 outer ring (Fig. 3 *B*) while helping to sustain TCR-triggered Ca^{2+} fluxes (Fig. 3 *C*). Therefore, we examined the accumulation of Kv1.3 within regions of interest defined by the CD2-CD58 corolla (CD58 region) and the LFA1-ICAM1 ring (ICAM1 region) (Fig. 5 *A*). These regions were defined dynamically in nascent synapses in early stages of formation (Fig. 5 *B*, top; Video S3) and mature synapses with ICAM1 rings and CD58 corollas (Fig. 5 *B*, bottom; Video S4) over time. Synaptic Kv1.3 was enriched within the CD2/CD58 corolla at the synapse and accumulated within this structure as it formed, reaching a 50% of total Kv1.3 present within the CD2/CD58 corolla in stablished mature synapses (Fig. 5 *C* and *D*). The dSMAC is also enriched with actin, and thus Kv1.3 also overlapped with the actin ring of the synapse (Fig. 5 *E*).

Kv1.3 is internalized at the cSMAC

As we could observe Kv1.3 accumulating in cytoplasmic compartments adjacent to the cSMAC (Fig. 1 *C* and *D*), we hypothesized that Kv1.3 was internalized at the cSMAC. Kv1.3 internalization is mediated by clathrin in other systems (28-30). We therefore analyzed the distribution of Kv1.3 and clathrin at different time points at the synaptic interface after SLB exposure. Kv1.3 steadily accumulated at the cSMAC (Fig. 6 *A* and *B*), which

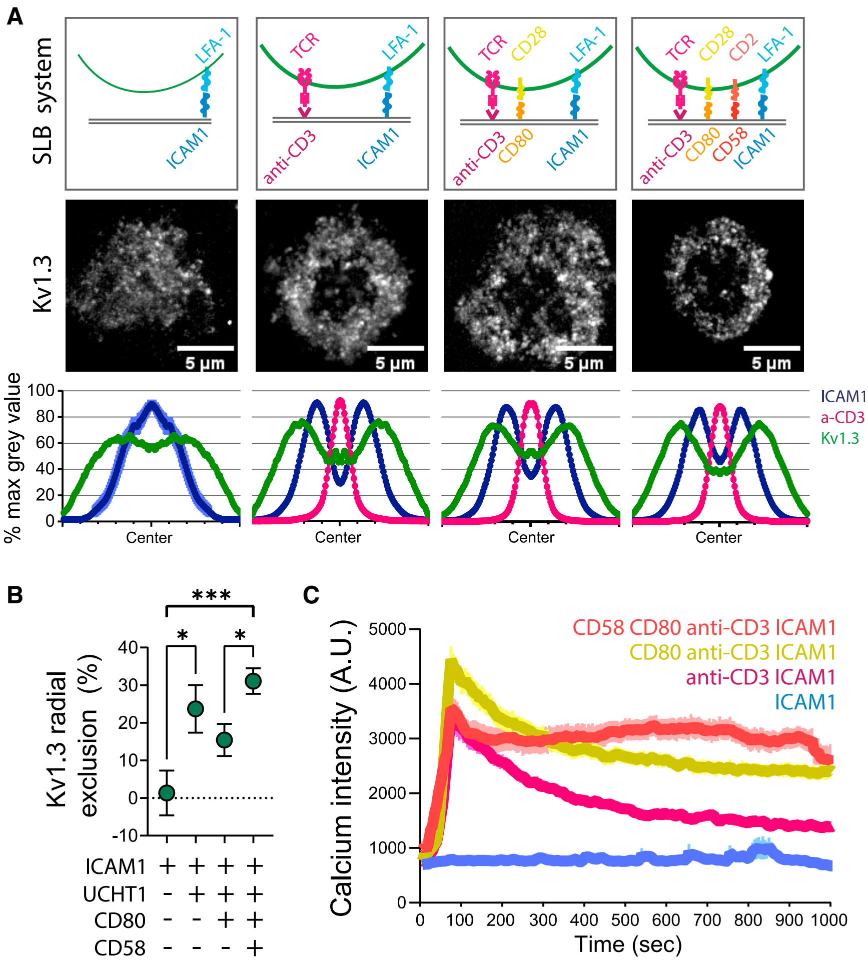

FIGURE 3 Effect of SLB composition on Kv1.3 localization and Ca^{2+} flux. (*A*) Top: schematics of the different SLB configurations tested. Middle: time projection from a representative TIRF video of Kv1.3 YFP (*gray*) expressed by CD4+ T cells exposed to the corresponding SLB. Bottom: histogram showing the average radial distribution of Kv1.3 (*green*), a-CD3 (*magenta*), and ICAM1 (*blue*). Data are the mean ± SE, n > 30 cells. (*B*) Quantification of Kv1.3 radial exclusion, computed as the percentage difference between the average Kv1.3 intensity in the central area and the distal area of the synapse. Data are the mean ± SE, n > 30 cells. $^{*}p < 0.05$, $^{***}p < 0.001$. One-way ANOVA test was used. (*C*) Average Ca2+ intensity over time triggered by live CD4+ T cells exposed to an SLB with the indicated composition (*blue*, ICAM1; *magenta*, anti-CD3 and ICAM1; *yellow*, CD80, anti-CD3, and ICAM1; *red*, CD58, CD80, anti-CD3, and ICAM1). Data are the mean ± SE, n = 20–60 cells. To see this figure in color, go online.

corresponded to a significant increase in Kv1.3 colocalization with clathrin (Fig. 6 *C*). In addition, Kv1.3 and clathrin accumulated in the synaptic cleft facing the plasma membrane from the cytoplasmic side of the cSMAC (Fig. 6 *D*), and Kv1.3 internalization was reduced by inhibitors of clathrin pathways (Fig. S2).

Kv1.3 is released in extracellular vesicles by activated CD4+ T cells

The TCR, together with other synaptic proteins, can be either internalized and released in exosomes or directly released in synaptic ectosomes at the cSMAC, both processes involving clathrin (31). Thus, we asked whether Kv1.3 is also incorporated in the EVs released by T cells. We purified the EVs from resting and activated human CD4+ T cells and analyzed their protein expression. The protein composition of the EVs was dependent on the activation state of the source cells. As previously described (32), EVs released by activated cells were enriched with the TCR and various other activation markers. Interestingly, we found that Kv1.3 was the main potassium channel released by $CD4^{+}$ T cells through EVs. In addition, like the TCR and other activation markers, Kv1.3 presence was highly enriched in EVs from activated $CD4^{+}$ T cells compared with EVs from resting $CD4^{+}$ T cells (Fig. 6 *E*; Table S1). In addition to Kv1.3, we also identified KCa3.1, which was also enriched in EVs from activated CD4+ T cells (Fig. 6 *E*). We further confirmed the presence of Kv1.3 in the EVs by dSTORM imaging of enriched EVs (Figs. 6 *F* and S3).

DISCUSSION

In this work, we show that Kv1.3, the main voltage-gated potassium channel in T cells, occupies the dSMAC of the immune synapse and forms into clusters that translocate to the cSMAC for internalization and release through EVs in activated $CD4^{+}$ T cells.

Previous work shows that Kv1.3 is mainly targeted to the IS by polarization of preexisting membrane-resident Kv1.3 channels (7). Interestingly, we show that Kv1.3 accumulation at the synaptic region was not homogeneous across the contact. TCR engagement triggered the localization of membrane-resident Kv1.3 to the dSMAC, with some Kv1.3 clusters accumulating at the cSMAC. Previous work shows that Kv1.3 has reduced mobility at the synaptic

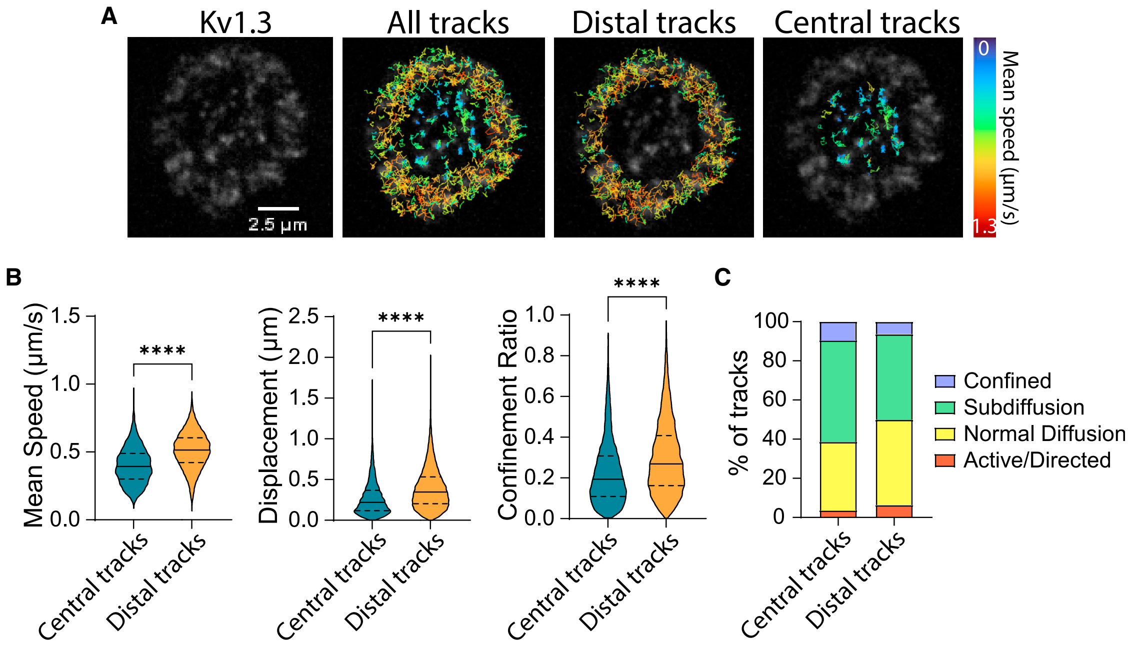

FIGURE 4 Kv1.3 clusters display reduced motion at the cSMAC. (*A*) Representative TIRF image of Kv1.3 YFP (*gray*) expressed by a CD4+ T cell forming a synapse with the SLB (a-CD3, ICAM1, CD80, and CD58). Synapses were imaged for 1 min at 0.3 s/frame, and SPT analysis was performed to analyze Kv1.3 trajectories. Maximum intensity projections over time were applied to the time frames and used to define the distal and central regions of interests (ROIs) based on the Kv1.3 ring. SPT analysis was performed for central and peripheral ROIs separately. Trajectory color indicates trajectory mean speed (µm/sec). (*B*) Violin plots showing track mean speed (mean of the link velocity (distance between the two spots divided by the time difference) over all the links of the track), total displacement (net distance traveled), and confinement ratio (net distance traveled divided by the total distance traveled) for trajectories within the synapse center or periphery. A total of 2955 tracks were analyzed as central tracks and 15,000 tracks as distal tracks from >60 cells. Student's *t*-test, $****p < 0.0001$. (*C*) Classification of trajectories based on the type of movement. To see this figure in color, go online.

contact compared with the rest of the plasma membrane (33). Our data provide further spatial resolution to this observation and show that Kv1.3 displays rapid diffusion in the dSMAC to effectively scan this area, whereas Kv1.3 clusters display more confined motion at the cSMAC.

To further understand the functional relevance of Kv1.3 segregation at the synapse, we aimed to identify the nature of the distal and central Kv1.3-containing compartments. Kv1.3 interacts with components of the actin cytoskeleton during the IS (34), and F-actin is enriched in the dSMAC and depleted in the central region of the synapse (35). Thus, we confirmed that the synaptic Kv1.3 localizes within the distal actin ring. Formation of the distal Kv1.3 ring was potentiated by activation CD2-CD58 corolla formation. In fact, Kv1.3 was enriched in the CD2-CD58 corolla as the synapse matured, with approximately half of the total Kv1.3 localizing there after 15 min. The CD2-CD58 corolla boosts signal to synergize with the TCR for an efficient T cell activation (2), and CD2 activation is required for sustained Ca^{2+} signaling (36,37). Formation of CD2 domains enhances recruitment of active Lck to the IS (38). Association of Lck and CD2 preferentially occurs within lipid raft microdomains after activation (39), and Kv1.3 also accumulates within raft-like structures during formation of the IS (25). Kv1.3 raft-targeting can be modulated by other regulating proteins highly expressed by T cells, such as the beta subunits (40,41). Kv1.3 was suggested to interact with Lck through the MAGUK protein Dlg1 in T cells (42). In Jurkat T cells, a constitutively active Lck reduces the PKA-dependent inhibition of Kv1.3 activity (43). Thus, Kv1.3 may localize in CD2 microclusters to assist Ca^{2+}-dependent pathways through links to active Lck. In addition, alterations in CD2/CD58 signaling are associated with multiple sclerosis (44) and rheumatoid arthritis (45). It is tempting to speculate that defects in the strength of CD2/CD58 interactions or the formation of the CD2/CD58 corolla could affect synaptic Kv1.3 organization and/or activity, thereby sensitizing the T cell to activation and leading to autoimmune responses. Indeed, formation of the Kv1.3 distal ring and sustenance of Ca^{2+} signal were simultaneously enhanced by CD2/CD58.

Previous work suggests that a mechanism to regulate Kv1.3 removal from the IS must exist, as the duration of Kv1.3 accumulation and redistribution outside the contact depends on the differentiation state of the T cell (7,17). The cSMAC is a region of active protein internalization (31) and release of vesicles (32,46,47). Therefore, it is likely that Kv1.3 encounters mediators of its internalization machinery at the cSMAC. The TCR is internalized at the cSMAC by a mechanism involving the clathrin machinery (31). Kv1.3 is also ubiquitinated and internalized by clathrin-mediated endocytosis, a process triggered by PKC

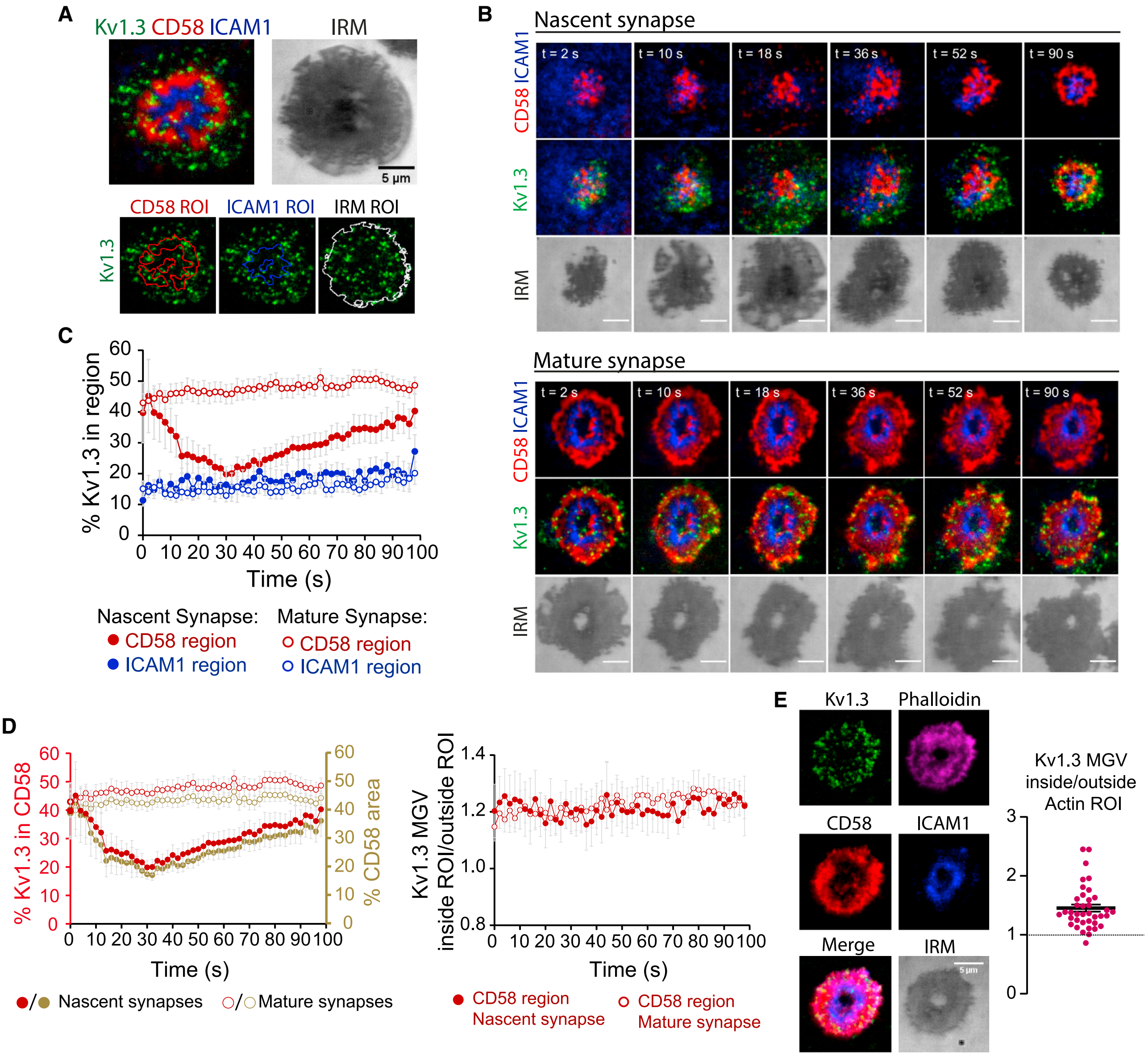

FIGURE 5 Kv1.3 is selectively recruited to the CD58 corolla at the synapse. (*A*) Representative TIRF image of a synapse formed between a CD4+ T cell (Kv1.3 YFP, *green*) and an SLB (CD58, *red*; ICAM1, *blue*; CD80, unlabeled; a-CD3, unlabeled). Videos were taken in live cells at 1.7 s/frame for 1.7 min. Bottom images summarize the analysis workflow: CD58, ICAM1, and IRM ROIs were segmented for each time frame, and the ROI was applied to the corresponding Kv1.3 image. Next, Kv1.3 intensity was measured within the CD58, ICAM1, or IRM ROI for each time point. (*B*) Representative TIRF time-lapse sequence showing a synapse as it is formed (nascent, contact to 120 s) and a synapse after ICAM1 ring formation (mature, >15 min). Scale bar represents 5 μm. (*C*) Quantification of the percentage Kv1.3 intensity inside CD58 (*red*) or ICAM1 (*blue*) ROIs with respect to the Kv1.3 intensity inside the IRM ROIs (whole cell contact). The analysis was performed separately on nascent (*solid dots*) and mature (*open dots*) synapses, using the analysis workflow summarized in (*A*). Data are the mean ± SE (n = 10 for nascent synapses and n = 20 for mature synapses). (*D*) Left plot: the percent Kv1.3 in the CD58 area (*left axis, red*) with the area fraction of CD58 in the synapse (*right axis, brown*). Right plot: Kv1.3 is continuously enriched in the CD58 regions in the nascent and mature synapse. (*E*) Kv1.3 enrichment at the F-actin ring as indicated by phalloidin staining in mature synapse. Plot shows the Kv1.3 mean intensity (MGV) inside versus outside of the actin ring. To see this figure in color, go online.

activation (29). In fact, PKCΘ is recruited to the IS to coordinate a complex signaling response to TCR engagement (48). Thus, we hypothesized that Kv1.3 is internalized at the cSMAC, together with other TCR components. We indeed observed an increase in the abundance of Kv1.3 over time at the cSMAC, which correlated with an increase in the Kv1.3-clathrin colocalization at the synapse. Interestingly, anti-CD3, which marks TCR-enriched vesicles, was segregated from the Kv1.3 signal in the cSMAC, which is consistent with TCR enrichment in synaptic ectosomes in contact with the SLB, whereas Kv1.3 may reside in exosomes released from multivesicular bodies after internalization

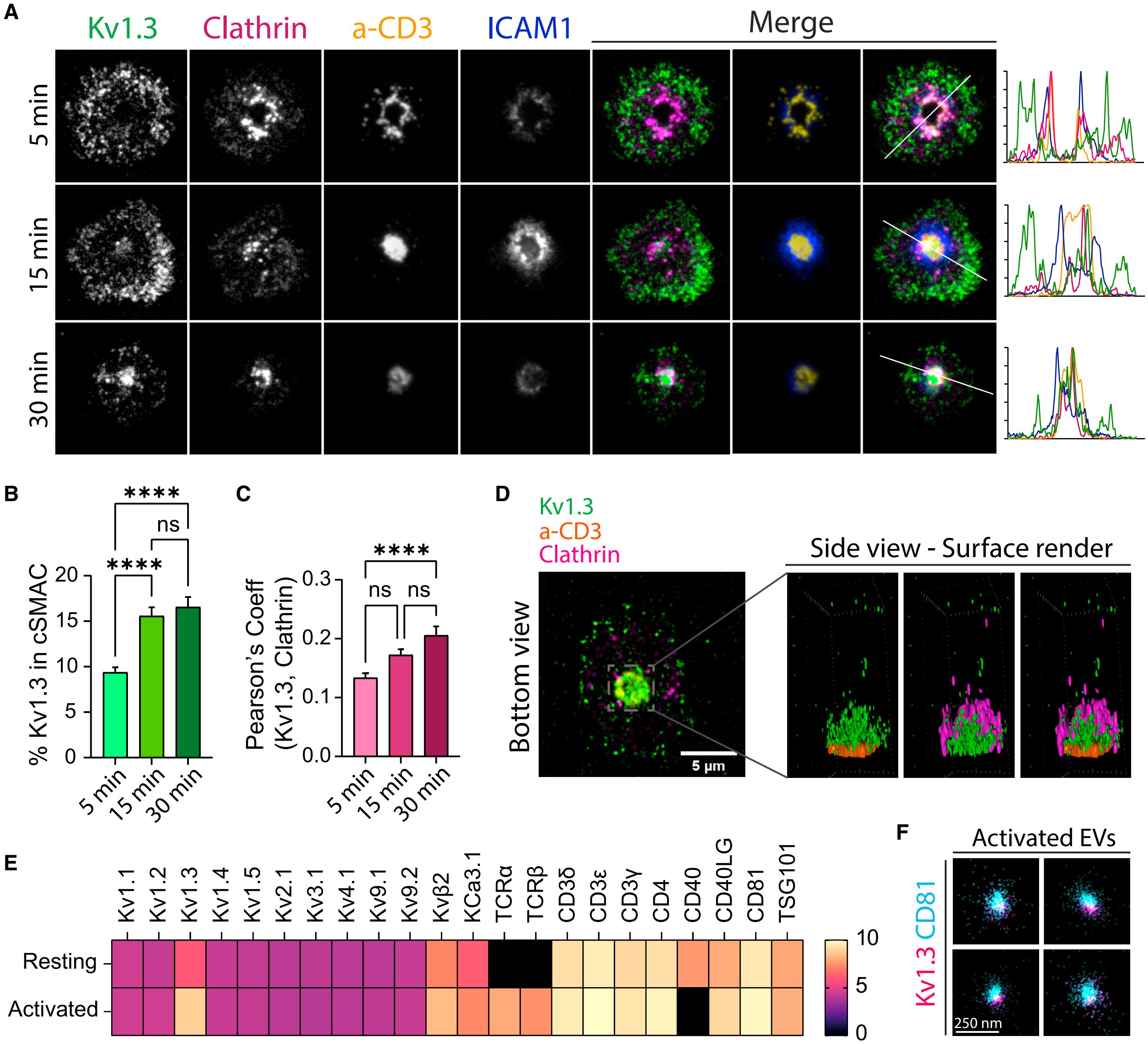

FIGURE 6 Kv1.3 is internalized at the cSMAC and released in extracellular vesicles. (*A*) Representative TIRF images of CD4+ T cells expressing Kv1.3 YFP and forming synapses with SLB (a-CD3 (*orange*), ICAM1 (blue), CD80 (unlabeled) and CD58 (unlabeled)). Cells were fixed after 5, 15, or 30 min after SLB exposure and immunostained against clathrin (*magenta*). Histograms show line profile of intensities. (*B*) Quantification of Kv1.3 abundance at the cSMAC (percentage Kv1.3 intensity in a-CD3 ROI respect total Kv1.3 intensity in the IRM ROI). a-CD3 intensity was used to define the cSMAC ROI. Data are the mean $\pm$ SE (n $>$ 55 cells). ****$p < 0.0001$ (one-way ANOVA). (*C*) Quantification of the Pearson's correlation coefficient between Kv1.3 and clathrin signals. Data are the mean $\pm$ SE (n $>$ 55 cells). ****$p < 0.0001$ (one-way ANOVA). (*D*) Representative image from a 3D Airyscan stack (*bottom view*) and surface render (*side view*) showing the intracellular region immediate to the cSMAC. (*E*) Heatmap showing the normalized expression of proteins identified in EVs from resting and activated T cells. All members of the potassium channels superfamily were interrogated, together with other activation markers and EVs markers. The heatmap shows all potassium channels detected. (*F*) Representative two-color dSTORM images of EV samples purified from activated CD4+ T cells expressing Kv1.3 YFP (*magenta*). CD81 (*cyan*) was used as an EV marker. To see this figure in color, go online.

through association with the clathrin-enriched zone at the plasma membrane ceiling of the synaptic cleft.

Finally, we show that Kv1.3 was specifically released in EVs from activated $CD4^+$ T cells. Our data suggest that the more static Kv1.3 clusters observed at the cSMAC might either be confined to endocytic pits or EVs. The choice between direct release in synaptic ectosomes or internalization followed by release in exosomes may be determined by the type of clathrin adapter that is recruited, HRS for synaptic ectosome budding or EPN1 for endocytosis, as recently reported for the TCR (31). Either mechanism of synaptic Kv1.3 downregulation (internalization or release) might serve as a negative feedback to terminate the channel activity and, therefore, Ca^{2+} signaling, in order to control T cell activation. In fact, previous work shows that the timing for Kv1.3 redistribution in and out of the IS depends on the

preactivation state of the T cells, as well as on their autoreactive phenotype, also suggesting that removal of Kv1.3 from the synaptic contact is important for proper T cell activation (17). It is tempting to speculate that Kv1.3-containing EVs released by activated T cells might be taken up by neighboring T cells. This could increase their Kv1.3 surface expression and sensitize them to activation, a potential mechanism for T cell quorum sensing. This hypothesis opens new avenues to understand the physiology of the immune response and will be interesting to follow up. In addition to Kv1.3, the Kv1.3-regulatory subunit Kvb2.1 (41) and the other main potassium channel Kca3.1 were also enriched in the EVs from activated CD4+ T cells.

To summarize, we show that during formation of the IS, Kv1.3 forms a distal ring that overlaps with the CD58/CD2 corolla, where it is well positioned to favor Ca^{2+} signaling and T cell activation. As the synapse matures, Kv1.3, together with other TCR components, is recruited to the cSMAC and internalized. This process likely reduces Kv1.3 function in sustaining Ca^{2+} entry, thereby promoting the termination of the early T cell activation. In addition, Kv1.3 is also released in EVs after activation. This might serve as an additional mechanism to terminate Kv1.3-dependent signals in the original cells while simultaneously sensitizing surrounding cells to activation.

This study improves our understanding of how ion channels are regulated during activation of the adaptive immune system and offers new insights into how these might be targeted by therapeutic interventions.

SUPPORTING MATERIAL

Supporting material can be found online at https://doi.org/10.1016/j.bpj.2023.08.011.

AUTHOR CONTRIBUTIONS

J.C., J.H.F., I.E., A.K., A.F., and M.L.D. designed research. J.C., A.K.J., M.N.-P., S.V., and P.D. performed research. J.C., A.K.J., and D.D. contributed analytic tools. J.C., A.K.J., and M.N.-P. analyzed data. J.C., A.F., and M.L.D. wrote the manuscript.

ACKNOWLEDGMENTS

We thank the essential support from Elke Kurz and Lina Chen, as well as the past and present members of the Felipe and Dustin laboratories. The authors also thank Raymond Moniz and Anish Suri for helpful discussions. We acknowledge the Kennedy Trust for Rheumatology Research, the Institute for Developmental and Regenerative Medicine, and Carl Zeiss GMBH for the microscopy facilities used in this research. We would also like to thank all the anonymized blood donors who contributed to our study. J.C. is a Cancer Research Institute Irvington Fellow supported by the CRI4503. J.C. was also supported by Fundación Tatiana Pérez de Guzmán el Bueno Fellowship and the Cue Biopharma Fellowship. A.K.J. was supported by the Clarendon Fund, University of Oxford; M.N.P. and I.E. by a MICINN Fellowship; P.D. by the Kennedy Trust Prize Studentship; D.D. by the European Commission ERC-2014-AdG_670930 to M.L.D.; A.K. by The Research Council of Norway in conjunction with Marie Sklodowska-Curie Actions 275466 and J.H.F. by a Sir Henry Wellcome Fellowship 107375/Z/15/Z. A.F. was supported by the Ministerio de Ciencia e Innovación (MICINN/AEI), Spain (PID2020-112647RB-I00 and 10.13039/501100011033) and European Regional Development Fund. M.L.D. was supported by the Wellcome PRF 100262Z/12/Z and the Kennedy Trust for Rheumatology Research (KTRR), Cell Dynamics Platform; KENN 20 21 17.

DECLARATION OF INTERESTS

Authors declare that they have no competing interests.

REFERENCES

1. Dustin, M. L. 2014. The immunological synapse. *Cancer Immunol. Res.* 2:1023–1033.
2. Demetriou, P., E. Abu-Shah, …, M. L. Dustin. 2020. A dynamic CD2-rich compartment at the outer edge of the immunological synapse boosts and integrates signals. *Nat. Immunol.* 21:1232–1243.
3. Trebak, M., and J. P. Kinet. 2019. Calcium signalling in T cells. *Nat. Rev. Immunol.* 19:154–169.
4. Feske, S., M. Prakriya, …, R. S. Lewis. 2005. A severe defect in CRAC Ca2+ channel activation and altered K+ channel gating in T cells from immunodeficient patients. *J. Exp. Med.* 202:651–662.
5. Cahalan, M. D., and K. G. Chandy. 2009. The functional network of ion channels in T lymphocytes. *Immunol. Rev.* 231:59–87.
6. Bose, T., A. Cieślar-Pobuda, and E. Wiechec. 2015. Role of ion channels in regulating Ca(2)(+) homeostasis during the interplay between immune and cancer cells. *Cell Death Dis.* 6:e1648.
7. Nicolaou, S. A., L. Neumeier, …, L. Conforti. 2009. Localization of Kv1.3 channels in the immunological synapse modulates the calcium response to antigen stimulation in T lymphocytes. *J. Immunol.* 183:6296–6302.
8. Chiang, E. Y., T. Li, …, J. L. Grogan. 2017. Potassium channels Kv1.3 and KCa3.1 cooperatively and compensatorily regulate antigen-specific memory T cell functions. *Nat. Commun.* 8, 14644.
9. Srivastava, S., L. Di, …, E. Y. Skolnik. 2009. The class II phosphatidylinositol 3 kinase C2beta is required for the activation of the K+ channel KCa3.1 and CD4 T-cells. *Mol. Biol. Cell.* 20:3783–3791.
10. Wulff, H., P. A. Calabresi, …, K. G. Chandy. 2003. The voltage-gated Kv1.3 K(+) channel in effector memory T cells as new target for MS. *J. Clin. Invest.* 111:1703–1713.
11. Rangaraju, S., V. Chi, …, K. G. Chandy. 2009. Kv1.3 potassium channels as a therapeutic target in multiple sclerosis. *Expert Opin. Ther. Targets.* 13:909–924.
12. Pérez-Verdaguer, M., J. Capera, …, A. Felipe. 2016. The voltage-gated potassium channel Kv1.3 is a promising multitherapeutic target against human pathologies. *Expert Opin. Ther. Targets.* 20:577–591.
13. Beeton, C., H. Wulff, …, K. G. Chandy. 2006. Kv1.3 channels are a therapeutic target for T cell-mediated autoimmune diseases. *Proc. Natl. Acad. Sci. USA.* 103:17414–17419.
14. Khodoun, M., A. A. Chimote, …, L. Conforti. 2020. Targeted knockdown of Kv1.3 channels in T lymphocytes corrects the disease manifestations associated with systemic lupus erythematosus. *Sci. Adv.* 6
15. Chandy, K. G., and R. S. Norton. 2017. Peptide blockers of Kv1.3 channels in T cells as therapeutics for autoimmune disease. *Curr. Opin. Chem. Biol.* 38:97–107.
16. Serrano-Albarrás, A., S. Cirera-Rocosa, …, A. Felipe. 2019. Fighting rheumatoid arthritis: Kv1.3 as a therapeutic target. *Biochem. Pharmacol.* 165:214–220.

17. Nicolaou, S. A., P. Szigligeti, …, L. Conforti. 2007. Altered dynamics of Kv1.3 channel compartmentalization in the immunological synapse in systemic lupus erythematosus. *J. Immunol.* 179:346–356.

18. Szilágyi, O., A. Boratkó, …, P. Hajdu. 2013. The role of PSD-95 in the rearrangement of Kv1.3 channels to the immunological synapse. *Pflugers Arch.* 465:1341–1353.

19. Vallejo-Gracia, A., D. Sastre, …, A. Felipe. 2021. KCNE4-dependent functional consequences of Kv1.3-related leukocyte physiology. *Sci. Rep.* 11, 14632.

20. Dustin, M. L., T. Starr, …, V. K. Thomas. 2007. Supported planar bilayers for study of the immunological synapse. *Curr. Protoc. Immunol.* Chapter 76:Unit 18 13.

21. Bolte, S., and F. P. Cordelières. 2006. A guided tour into subcellular colocalization analysis in light microscopy. *J. Microsc.* 224:213–232.

22. Felce, J. H., L. Parolini, …, M. L. Dustin. 2020. Single-Molecule, Super-Resolution, and Functional Analysis of G Protein-Coupled Receptor Behavior Within the T Cell Immunological Synapse. *Front. Cell Dev. Biol.* 8, 608484.

23. Tinevez, J. Y., N. Perry, …, K. W. Eliceiri. 2017. TrackMate: An open and extensible platform for single-particle tracking. *Methods.* 115:80–90.

24. Wagner, T., A. Kroll, …, M. Wiemann. 2017. Classification and Segmentation of Nanoparticle Diffusion Trajectories in Cellular Micro Environments. *PLoS One.* 12, e0170165.

25. Panyi, G., G. Vámosi, …, S. Damjanovich. 2004. Kv1.3 potassium channels are localized in the immunological synapse formed between cytotoxic and target cells. *Proc. Natl. Acad. Sci. USA.* 101:1285–1290.

26. Fung-Leung, W. P., W. Edwards, …, A. D. Wickenden. 2017. T Cell Subset and Stimulation Strength-Dependent Modulation of T Cell Activation by Kv1.3 Blockers. *PLoS One.* 12, e0170102.

27. DeCoursey, T. E., K. G. Chandy, …, M. D. Cahalan. 1984. Voltage-gated K+ channels in human T lymphocytes: a role in mitogenesis? *Nature.* 307:465–468.

28. Martínez-Mármol, R., N. Comes, …, A. Felipe. 2016. Unconventional EGF-induced ERK1/2-mediated Kv1.3 endocytosis. *Cell. Mol. Life Sci.* 73:1515–1528.

29. Martínez-Mármol, R., K. Styrczewska, …, A. Felipe. 2017. Ubiquitination mediates Kv1.3 endocytosis as a mechanism for protein kinase C-dependent modulation. *Sci. Rep.* 7, 42395.

30. Estadella, I., O. Pedrós-Gámez, …, A. Felipe. 2020. Endocytosis: A Turnover Mechanism Controlling Ion Channel Function. *Cells.* 9, 1833.

31. Kvalvaag, A., S. Valvo, …, M. L. Dustin. 2023. Clathrin mediates both internalization and vesicular release of triggered T cell receptor at the immunological synapse. *Proc. Natl. Acad. Sci. USA.* 120, e2211368120.

32. Saliba, D. G., P. F. Céspedes-Donoso, …, M. L. Dustin. 2019. Composition and structure of synaptic ectosomes exporting antigen receptor linked to functional CD40 ligand from helper T cells. *Elife.* 8, e47528.

33. Sebestyén, V., É. Nagy, …, G. Vámosi. 2022. Role of C-Terminal Domain and Membrane Potential in the Mobility of Kv1.3 Channels in Immune Synapse Forming T Cells. *Int. J. Mol. Sci.* 23:3313.

34. Hajdu, P., G. V. Martin, …, L. Conforti. 2015. The C-terminus SH3-binding domain of Kv1.3 is required for the actin-mediated immobilization of the channel via cortactin. *Mol. Biol. Cell.* 26:1640–1651.

35. Fritzsche, M., R. A. Fernandes, …, C. Eggeling. 2017. Cytoskeletal actin dynamics shape a ramifying actin network underpinning immunological synapse formation. *Sci. Adv.* 3, e1603032.

36. Espagnolle, N., D. Depoil, …, S. Valitutti. 2007. CD2 and TCR synergize for the activation of phospholipase Cgamma1/calcium pathway at the immunological synapse. *Int. Immunol.* 19:239–248.

37. Dustin, M. L., D. Olive, and T. A. Springer. 1989. Correlation of CD2 binding and functional properties of multimeric and monomeric lymphocyte function-associated antigen 3. *J. Exp. Med.* 169:503–517.

38. Kaizuka, Y., A. D. Douglass, …, R. D. Vale. 2009. The coreceptor CD2 uses plasma membrane microdomains to transduce signals in T cells. *J. Cell Biol.* 185:521–534.

39. Nunes, R. J., M. A. A. Castro, …, A. M. Carmo. 2008. Protein interactions between CD2 and Lck are required for the lipid raft distribution of CD2. *J. Immunol.* 180:988–997.

40. Roig, S. R., S. Cassinelli, …, A. Felipe. 2022. Oligomerization and Spatial Distribution of Kvbeta1.1 and Kvbeta2.1 Regulatory Subunits. *Front. Physiol.* 13, 930769.

41. Roig, S. R., S. Cassinelli, …, A. Felipe. 2022. S-acylation-dependent membrane microdomain localization of the regulatory Kvbeta2.1 subunit. *Cell. Mol. Life Sci.* 79:230.

42. Hanada, T., L. Lin, …, A. H. Chishti. 1997. Human homologue of the Drosophila discs large tumor suppressor binds to p56lck tyrosine kinase and Shaker type Kv1.3 potassium channel in T lymphocytes. *J. Biol. Chem.* 272:26899–26904.

43. Kuras, Z., V. Kucher, …, L. Conforti. 2012. Modulation of Kv1.3 channels by protein kinase A I in T lymphocytes is mediated by the disc large 1-tyrosine kinase Lck complex. *Am. J. Physiol. Cell Physiol.* 302:C1504–C1512.

44. De Jager, P. L., C. Baecher-Allan, …, D. A. Hafler. 2009. The role of the CD58 locus in multiple sclerosis. *Proc. Natl. Acad. Sci. USA.* 106:5264–5269.

45. Raychaudhuri, S., B. P. Thomson, …, R. M. Plenge. 2009. Genetic variants at CD28, PRDM1 and CD2/CD58 are associated with rheumatoid arthritis risk. *Nat. Genet.* 41:1313–1318.

46. Dustin, M. L., and K. Choudhuri. 2016. Signaling and Polarized Communication Across the T Cell Immunological Synapse. *Annu. Rev. Cell Dev. Biol.* 32:303–325.

47. Céspedes, P. F., A. Jainarayanan, …, M. L. Dustin. 2022. T-cell trans-synaptic vesicles are distinct and carry greater effector content than constitutive extracellular vesicles. *Nat. Commun.* 13:3460.

48. Zanin-Zhorov, A., M. L. Dustin, and B. R. Blazar. 2011. PKC-theta function at the immunological synapse: prospects for therapeutic targeting. *Trends Immunol.* 32:358–363.

Biophysical Journal, Volume *123*

Supplemental information

Dynamics and spatial organization of Kv1.3 at the immunological synapse of human CD4+ T cells

Jesusa Capera, Ashwin Jainarayanan, María Navarro-Pérez, Salvatore Valvo, Philippos Demetriou, David Depoil, Irene Estadella, Audun Kvalvaag, James H. Felce, Antonio Felipe, and Michael L. Dustin

SUPPLEMENTAL FIGURES:

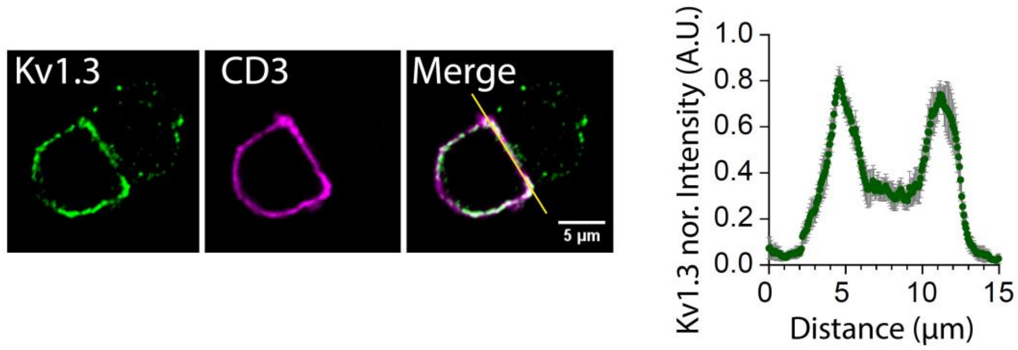

Figure S1*. Kv1.3 accumulates as a distal ring in the case of T cell-APC conjugates*. Representative confocal image showing a conjugate between a human CD4+ T cell and a Raji B cell. Endogenous Kv1.3 and CD3 molecules were immunostained (green and magenta, respectively). A line (yellow in the merged image) was defined across the synaptic contact and Kv1.3 intensity. Plot shows the average Kv1.3 intensity across the line (n=10 cells).

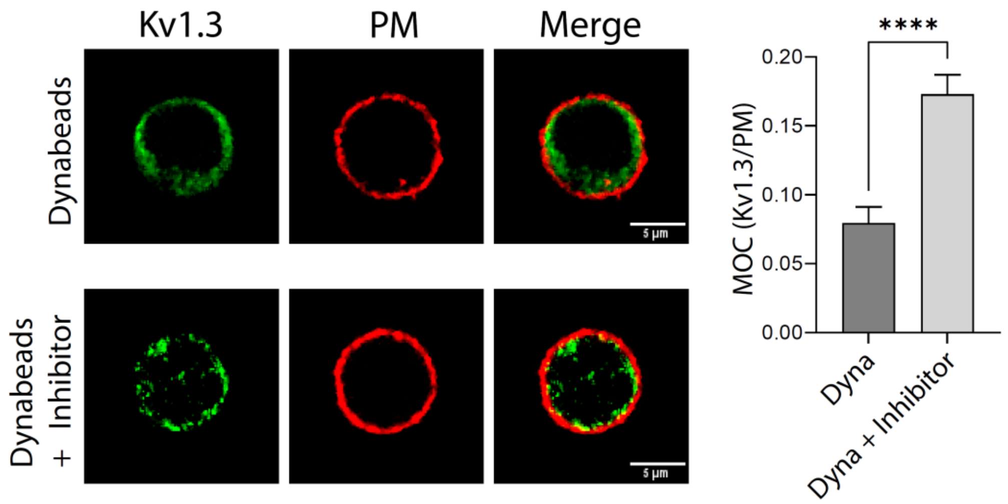

Figure S2*. Kv1.3 internalization after activation*. Representative confocal images showing a a human CD4+ T cell after 15 min treatment with anti-CD3/CD28 Dynabeads (Gibco, 1:1 cell-to Dynabead ratio). Dynabead treatment was performed with or without 5 µg/ml of Chlorpromazine. Plot shows the Manders Coefficient between Kv1.3 and the plasma membrane marker. Data is the mean ± SE (n=30 cells, T-test).

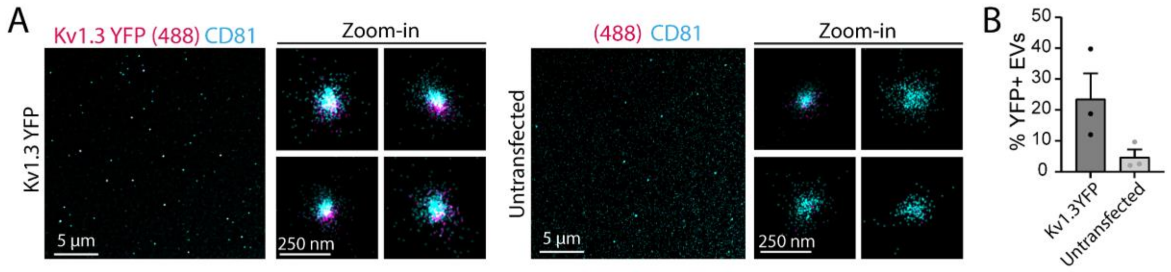

Figure S3. *Kv1.3 is released in extracellular vesicles (EVs) from activated CD4⁺ T cells.* **(A)** Representative dSTORM images showing EVs from activated CD4⁺ T cells transfected or not with Kv1.3-YFP and immunostained for CD81 (EV marker). Zoom in images show representative examples of individual EVs. **(B)** Quantification of the percentage of YFP positive EVs. EVs were segmented using the CD81 signal and YFP mean intensity was quantified for each EV. The mean YFP intensity for the untransfected sample was used to threshold for positive YFP particles. Data is the mean ± SE (n=3).

	Raw Intensity		Log10 transformed Intersity	
	Resting EVs	Activated EVs	Resting EVs	Activated EVs
KCNA1	31121	34121	4.493053544	4.533021751
KCNA2	21531	32193	4.333064201	4.50776145
KCNA3	1053100	827300000	6.022469613	8.917663024
KCNA4	21320	21231	4.3287872	4.32697045
KCNA5	17411	19821	4.240823716	4.297125562
KCNB1	12311	11311	4.090293331	4.053501002
KCNC1	14130	11130	4.150142162	4.046495164
KCND1	11643	13243	4.066064897	4.121986379
KCNS1	11212	14402	4.049683089	4.158422807
KCNS2	21113	22311	4.324549948	4.348519036
KCNAB2	13920000	246370000	7.143639235	8.391587824
KCNN4	1630600	19324000	6.212347438	7.286097029
TCRA	1	98024000	0	7.991332421
TCRB	1	30443000	0	7.483487448
CD3D	1805200000	3768200000	9.256525325	9.576133945
CD3E	4244000000	1.0516E+10	9.627775375	10.02185058
CD3G	1185700000	2535600000	9.07397482	9.404080743
CD4	1920700000	5881600000	9.283459536	9.769495485
CD40	43670000	1	7.640183192	0
CD40LG;TNFSF5	104820000	1283200000	8.020444155	9.108294351
CD81	3174400000	7498300000	9.50166165	9.874962812
TSG101	79443000	138660000	7.900055636	8.141951196

Table 1. Intensities (raw and log10 transformed values) of the molecules present in 1 ml of (10e8 EV/ml) of purified EVs from resting and activated CD4+ T cells.
